# Milk Fat Globule Membrane Supplementation in Formula-fed Rat Pups Improves Reflex Development and May Alter Brain Lipid Composition

**DOI:** 10.1038/s41598-018-33603-8

**Published:** 2018-10-15

**Authors:** Sara Moukarzel, Roger A. Dyer, Cyrielle Garcia, Alejandra M. Wiedeman, Guilaine Boyce, Joanne Weinberg, Bernd O. Keller, Rajavel Elango, Sheila M. Innis

**Affiliations:** 1 Department of Pediatrics and the Larsson-Rosenquist Foundation Mother-Milk-Infant Center of Research Excellence, University of California San Diego, La Jolla, CA 92093 USA; 2 Analytical Core for Metabolomics and Nutrition (ACMaN), BC Children’s Hospital Research Institute, Vancouver, BC V5Z 4H4 Canada; 3 Department of Cellular & Physiological Sciences, University of British Columbia, Vancouver, BC V6T 1Z3 Canada; 4 Department of Pediatrics, University of British Columbia, Vancouver, BC V5Z 3V4 Canada; 5 School of Population and Public Health, University of British Columbia, Vancouver, BC V6T 1Z3 Canada

## Abstract

Human milk contains nutritional, immunoprotective and developmental components that support optimal infant growth and development. The milk fat globule membrane (MFGM) is one unique component, comprised of a tri-layer of polar lipids, glycolipids, and proteins, that may be important for brain development. MFGM is not present in most infant formulas. We tested the effects of bovine MFGM supplementation on reflex development and on brain lipid and metabolite composition in rats using the “pup in a cup” model. From postnatal d5 to d18, rats received either formula supplemented with MFGM or a standard formula without MFGM; a group of mother-reared animals was used as reference/control condition. Body and brain weights did not differ between groups. MFGM supplementation reduced the gap in maturation age between mother-reared and standard formula-fed groups for the ear and eyelid twitch, negative geotaxis and cliff avoidance reflexes. Statistically significant differences in brain phospholipid and metabolite composition were found at d13 and/or d18 between mother-reared and standard formula-fed groups, including a higher phosphatidylcholine:phosphatidylethanolamine ratio, and higher phosphatidylserine, glycerol-3 phosphate, and glutamine in mother-reared compared to formula-fed pups. Adding MFGM to formula narrowed these differences. Our study demonstrates that addition of bovine MFGM to formula promotes reflex development and alters brain phospholipid and metabolite composition. Changes in brain lipid metabolism and their potential functional implications for neurodevelopment need to be further investigated in future studies.

## Introduction

Suboptimal provision of nutrients during early postnatal development may have lasting consequences on the brain that range from structural changes to subtle effects on neural functioning^1^. Human milk promotes optimal brain development and is considered the gold standard for feeding infants. Substitutes for human milk (infant formula), which differ in composition from human milk, become the only source of nutrition in situations when human milk is not available or sufficient in volume. It is therefore important to understand which and how human milk components, or lack thereof, impact brain development.

Lipids in the human milk aqueous phase consist of globules formed of a triacylglycerol core surrounded by a unique membrane, the milk fat globule membrane (MFGM)^2^. Unlike any other biological membrane, the MFGM is comprised of three layers of potentially bioactive molecules including polar lipids (i.e, phosphatidylcholine, phosphatidylethanolamine, sphingomyelin, plasmalogens, gangliosides), cholesterol, proteins and glycoproteins^3,4^. The majority of infant formula products, however, do not contain MFGM, and their lipid constituents are derived from vegetable oils which lack the bioactive lipid molecules present in MFGM^5^. Interest in the biological benefits of MFGM has greatly increased over the last five years, largely driven by the recent commercial availability of bovine MFGM and its potential use as a functional dietary ingredient in infant formula.

Earlier animal studies suggest that individual MFGM components may have positive effects on structural and functional brain development, as previously reviewed^6,7^. More recently and among other clinical trials^8^, infants fed a low-protein low-energy formula with added bovine MFGM from 2 to 6 months scored significantly higher on cognitive testing at 12 months using the Bayley Scales of Infant and Toddler Development than infants fed standard formula, with scores of the former being similar to those of a reference group of breastfed infants^9^. While mechanisms of action remain unclear, MFGM may have direct effects on the brain (e.g. provision of substrates for brain structural development), indirect effects via gut development and the gut-brain axis, or other unknown mechanisms^10–12^. Our research collaborators have recently shown that the addition of MFGM to formula restored normal development of the gut epithelium and microbiome in artificially-reared neonatal rats^13^. An increase in brain phospholipids and a characteristic decrease in brain phosphatidylcholine:phosphatidylethanolamine (PC:PE) ratio are known neurodevelopmental changes with implications for brain function^14–20^. Additionally, the long-chain polyunsaturated fatty acid docosahexaenoic acid (DHA) increases in the rat brain during postnatal development similar to the human and is primarily present in gray matter PE and phosphatidylserine (PS)^14,18–21^. Our objectives for the current study were to: (1) determine whether feeding MFGM improves the development of physical features and reflexes in artificially-reared rat pups; and (2) begin to elucidate potential mechanisms behind possible improved neurodevelopment with MFGM feeding.

## Results

### Rat pup growth, physical features and reflex development

**Figure 1:**
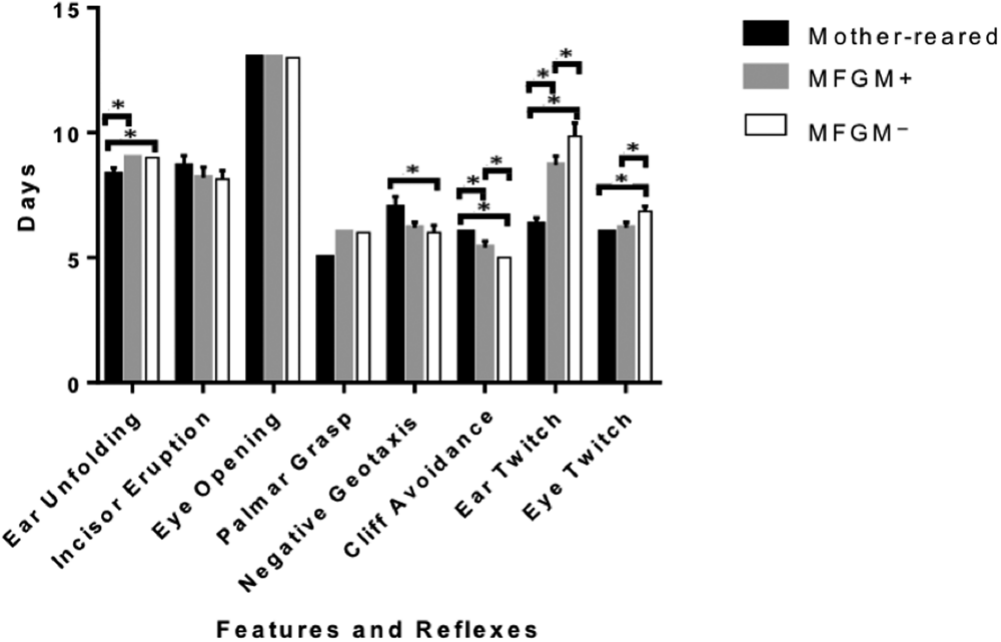
Black, gray and white bars correspond to the mother-reared, MFGM+ group and MFGM− group respectively.

Mother-reared pups, pups fed formulas with MFGM (MFGM+ group), and pups fed formula without MFGM (MFGM− group) had similar body and brain weights at all time points (Supplementary Table 1). As maturation of selected physical features and development of reflexes are markers of neurodevelopment^22^, we assessed if maturation age (i.e, day on which a milestone is achieved) was impacted by MFGM supplementation. The average age at which the eyes opened and the incisors erupted did not differ among the three groups (Fig. 1 and Supplementary Table 2). Addition of MFGM to formula did not affect maturation age for ear unfolding, which occurred significantly earlier in mother-reared pups (*P* = 0.001). The negative geotaxis reflex occurred significantly earlier in the MFGM− group compared to the mother-reared group (*P* = 0.015) for which the maturation age was closer to pups in the MFGM+ group (*P* = 0.069). Maturation age for cliff avoidance and ear twitch reflexes were significantly different across the three groups (*P* < 0.001), and for both tests, the MFGM+ group was closer to the mother-reared group compared to the MFGM− group. Eyelid twitch was significantly delayed in the MFGM− group compared to the mother-reared group (*P* < 0.001), with no significant difference between the mother-reared and MFGM+ groups.

### Changes in selected brain lipids

**Figure 2:**
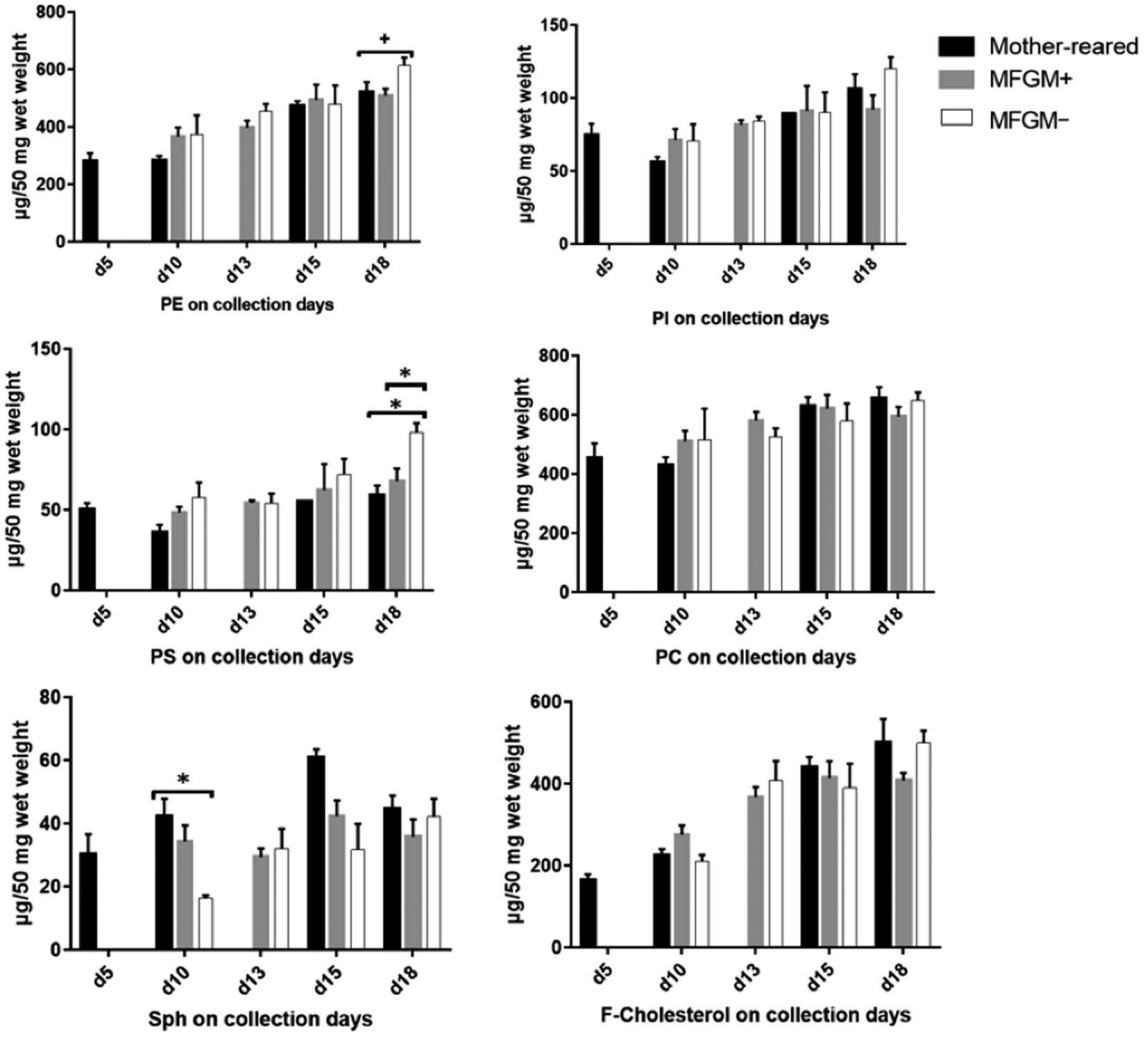
Black, gray, and white bars correspond to the mother-reared, MFGM+ group and MFGM− group respectively.

At d10, we found significantly lower sphingomyelin (Sph) in the MFGM− group compared to the mother-reared group (*P* = 0.015), but no difference in Sph between the mother-reared and MFGM+ groups or between the MFGM+ and MFGM− groups (Fig. 2 and Supplementary Table 3). At d18, the brain lipid composition of the MFGM+ group was closer to that of the mother-reared group compared to MFGM−. More specifically, PS which was not significantly different between MFGM+ and mother-reared groups, was significantly higher in the MFGM− group compared to the mother-reared group (*P* = 0.001) and compared to the MFGM+ group (*P* = 0.005). Additionally, there was a strong trend for a significant difference in PE across the three groups at d18 (*P* = 0.052), with higher PE in the MFGM− group compared to the others. As well, at d18, the PC:PE ratio was significantly lower in the MFGM− group compared to the mother-reared group, and feeding MFGM brought the PC:PE ratio closer (but still with a statistically significant difference) to that of the mother-reared group (Supplementary Table 3). Therefore, feeding MFGM to formula-fed infant rats appears to reduce the difference in brain lipid class composition between formula-fed and mother-reared rats.

Next, we focused our fatty acid analysis on differences in DHA (mg/100 mg fatty acids) in brain PE among the groups. The low concentrations of PS in the brain samples hindered our ability to accurately measure fatty acid composition, and we hope other investigators will be interested in addressing this gap in the literature. DHA in PE was remarkably similar in the MFGM+ and MFGM− groups at d10, d15 and d18, and both were significantly lower than in the mother-reared group at d10 and d18 (Table 1). We found significantly lower DHA in the MFGM+ group compared to the MFGM− group at d13 (*P* = 0.003), and the MFGM+ group was instead significantly higher in arachidonic acid (20:4 n − 6) (*P* = 0.001).

**Table 1:** Polyunsaturated fatty acid composition of brain PE of mother-reared rats and experimental rats fed formula with or without MFGM.

	Mother-reared	MFGM+	MFGM−	Mother-reared	MFGM+	MFGM−
	**d10**			**d13**		
18:2 ω-6	1.07 ± 0.37	0.48 ± 0.34	1.20 ± 1.33	NA	0.87 ± 0.09^a^	1.68 ± 0.78^b^
20:4 ω-6	21.0 ± 1.24	22.1 ± 2.37	22.8 ± 1.44	NA	21.6 ± 0.29^a^	18.8 ± 1.30^b^
22:4 ω-6	4.80 ± 0.42^a^	5.06 ± 0.43^a^	6.02 ± 0.31^b^	NA	5.89 ± 0.49	6.00 ± 0.47
22:5 ω-6	2.10 ± 0.12	2.98 ± 0.18	3.66 ± 0.44	NA	3.09 ± 0.26	2.89 ± 0.50
20:5 ω-3	0.08 ± 0.01	0.10 ± 0.04	0.05 ± 0.04	NA	0.19 ± 0.25	0.08 ± 0.03
22:5 ω-3	0.56 ± 0.04^a^	0.35 ± 0.04^b^	0.34 ± 0.06^b^	NA	0.33 ± 0.05	0.32 ± 0.01
22:6 ω-3	19.9 ± 0.9^a^	16.4 ± 1.14^b^	16.4 ± 2.85^b^	NA	16.0 ± 0.77^a^	17.4 ± 0.46^b^
	**d15**			**d18**		
18:2 ω-6	1.97 ± 0.33	1.37 ± 0.68	2.45 ± 1.17	1.67 ± 0.01	1.66 ± 0.45	2.22 ± 0.19
20:4 ω-6	19.1 ± 0.74	21.3 ± 1.00	21.4 ± 1.99	19.3 ± 1.48	20.2 ± 1.24	20.6 ± 1.03
22:4 ω-6	5.53 ± 0.45	6.20 ± 0.58	6.43 ± 1.01	6.10 ± 0.34	6.20 ± 0.11	6.51 ± 0.21
22:5 ω-6	1.55 ± 0.36^a^	3.41 ± 0.62^b^	3.94 ± 1.03^b^	1.35 ± 0.04	2.51 ± 0.32	2.27 ± 1.33
20:5 ω-3	0.13 ± 0.08	0.08 ± 0.08	0.09 ± 0.01	0.07 ± 0.01	0.09 ± 0.01	0.05 ± 0.03
22:5 ω-3	0.54 ± 0.15	0.40 ± 0.05	0.36 ± 0.05^*^	0.43 ± 0.03^a^	0.32 ± 0.01^b^	0.32 ± 0.03^b^
22:6 ω-3	18.2 ± 2.14	16.1 ± 2.57	15.6 ± 1.77	19.6 ± 0.65^a^	16.7 ± 0.44^b^	16.4 ± 1.15^b^

Values are for mg/100 mg fatty acids and are mean ± SD; n = 5–6 per group per time point. Values within a row at any particular time point with different letters are significantly different from each other, using ANOVA followed by post hoc LSD test (*P* < 0.05). *Difference tends to be significantly different across groups (*P* = 0.064).

### Changes in brain tissue metabolites

No significant differences in brain metabolites (mass spectra were acquired at m/z between 75–650) were found at d10 postnatal among the three groups. There were generally limited differences in brain metabolites at d13, d15, and d18 postnatal (Table 2 and Supplementary Table 4). At d13, eight metabolites were significantly decreased in the MFGM+ group compared to MFGM−; metabolites in the MFGM− group were 1.4 to 3.5 folds higher than in the MFGM+ group. At d15, brain samples from the mother-reared group were significantly lower in threonine, glycine, glutamine and alanine compared to both formula-fed groups, independent of whether or not MFGM was added to the formula. The only difference related to MFGM feeding was in inositol, which was significantly lower in the mother-reared group compared to MFGM− but not different between the mother-reared and MFGM+ groups. At d18, threonine, inositol, and glycerol-3-phosphate (G-3-P) did not differ between the mother-reared and MFGM+ groups but were significantly increased in MFGM−. While glutamine was significantly different across the three groups (*P* = 0.048), area counts for glutamine in the MFGM+ brain samples were closer to those of mother-reared rats compared to MFGM−.

**Table 2:** Significant differences in metabolite area counts between mother-reared rats and experimental rats fed a diet with or without MFGM.

	Mother-reared *(n* = 6)	MFGM+ (*n* = 7)	MFGM− (*n* = 7)
**At day 13**			
Lactate		74 ± 6.1	100 ± 18
Serine		3.8 ± 2.1	7.7 ± 2.2
Aspartate		16.4 ± 3.5	2.6 ± 0.5
Glutamine	NA	59 ± 22	97 ± 19
Glycerol-3-Phosphate		4.4 ± 2.4	15 ± 6.6
Glycine		3.2 ± 0.5	6.1 ± 0.7
Inositol		57 ± 2.3	160 ± 55
Threonine		4.9 ± 1.9	140 ± 7.6
**At day 15**			
Threonine	4.3 ± 0.60^a^	19 ± 6.6^b^	20 ± 9.3^b^
Glycine	3.5 ± 0.22^a^	7.1 ± 2.4^b^	6.6 ± 2.6^b^
Glutamine	60 ± 9.0^a^	110 ± 28^b^	100 ± 24^b^
Alanine	4.4 ± 0.57^a^	120 ± 4.0^b^	120 ± 5.9^b^
Inositol	120 ± 130^a^	200 ± 6.2^a,b^	210 ± 38^b^
**At day 18**			
Threonine	8.3 ± 2.5^a^	15 ± 8.2^a^	24 ± 8.6^b^
Glutamine	71 ± 35^a^	110 ± 22^b^	140 ± 47^c^
Inositol	140 ± 47^a^	220 ± 47^a^	270 ± 30^b^
Glycerol-3-phosphate	24 ± 11^a^	27 ± 3^a^	76 ± 2.6^b^

Values are GC-MS area counts ×10^6^ as mean ± SD; n = 5–6 per group per time point. NA, not available data. Values within a row at any particular time point with different letters are significantly different from each other, using ANOVA followed by post hoc LSD test for three-group comparisons (*P* < 0.05). ANOVA was used to compare MFGM+ compared to MFGM− groups at d13 and only metabolites with significant differences between the groups are shown.

## Discussion

Human milk is a complex biological fluid that has evolved over 150 million years to be a crucial source of not only nutrients but also immunoprotective and development-promoting bioactive compounds to the infant^7^. In situations when human milk is not available, it is important to ensure infant formula is as close to human milk composition as possible. Infant formula products commonly contain lipids from vegetable oil mixtures that lack MFGM. Over the last two years, formula with added bovine MFGM, which contains components similar and close in composition to human MFGM^23–25^, has become available in China and the United States. The addition of bovine MFGM has been justified in part by findings from intervention studies in term infants, which suggest bovine MFGM supplementation can narrow the gap between formula-fed and breastfed infants with respect to neurodevelopment^8,9,26^. In this study, we examined the effect of bovine MFGM supplementation in formula on reflex development and explored changes in development-sensitive brain lipids and metabolites using the rat “pup in a cup” artificial rearing model.

Somatic maturation and reflex development during the first two weeks postnatally are good indicators of the functional maturity of the brain during early development^22,27^. We found that MFGM supplementation reduced the gap in maturation age for few reflexes (ear and eyelid twitch, and cliff avoidance) between mother-reared and formula-fed controls. Reflex outcomes in response to MFGM supplementation have not yet been reported in humans. However, formula-fed infants supplemented with the same bovine MFGM product used in our experiments (Lacprodan MFGM-10; Arla Foods Ingredients) between ~2–6 months of age had significantly closer cognitive scores to those of breastfed infants compared to non-supplemented infants^9^. Cognitive scores were based on the Bayley Scales of Infant Development, 3^rd^ edition, and assessment was done at 12 months of age. Contrary to our findings on sensory reflex and negative geotaxis tests, Gurnida *et al*.^26^ reported significantly higher hand and eye coordination IQ scores in infants consuming formula with added complex lipids, derived from bovine MFGM, compared to standard formula-fed infants. Measurements of hand and eye coordination are one way to assess adequate motor coordination. The latter not only requires adequate musculoskeletal functioning, but also adequate sensory, vestibular, and proprioceptive functioning (i.e, ability to balance and sense body movement), which are collectively assessed using the negative geotaxis test^28,29^. For cliff avoidance, it is challenging to interpret or speculate why both formula-fed groups (with or without MFGM) had an accelerated reflex development compared to mother-reared animals. Accelerated achievement of developmental milestones has been shown to occur due to prenatal exposure to selective serotonin reuptake inhibitors and to alter the trajectory for language development unfavorably in infants^30^. However, since non-supplemented animals successfully completed the cliff avoidance test on day 5, which is the cannulation day and first day of feeding, it is questionable whether differences in maturation age across groups for cliff avoidance are explained by diet. Additionally, artificially-reared pups might be more acclimatized to human handling, more adaptive to environments outside the dam cages, and may be less fearful in exploring the cliff avoidance test area^31,32^. Findings from the collective assessment of reflexes in this study highlight that effects of MFGM on reflex development are not comprehensive (not for all reflexes) and more research is needed to determine what the functional implications of the significant differences are for brain function. It is known for other more -studied nutrients that effects on brain development may be specific to particular cognitive domains, such as the effect of DHA intake on language and short-term memory but not visual-motor coordination in children, as we have recently reported^33^.

In addition to the impact of MFGM supplementation on early reflex development, we were interested in differences in major lipid species and fatty acids in the brain during the period of exclusive milk or formula feeding; hence we analyzed brain samples across different time points up to postnatal d18. While typical weaning age of mother-reared rats is around d21 postnatal^34^, we noticed during our experimental protocol pilot stage that artificially reared rats start chewing on the feeding tubes after d18 resulting in formula leakage and inaccurate assessment of the milk volume consumed. Because this work was the first to explore differences in brain lipids due to MFGM feeding in artificially-reared rats, we selected as many timepoints as logistically possible between cannulation day and weaning to explore potential effects on brain lipid composition. In the rat, consistent with the peak in human synaptogenesis from 34 wk gestation-24 months postnatal, a synaptogenesis surge occurs during the second week postnatal, between time points d13 and d18 in our study^35^. This results in the proportion of PC decreasing with a concurrent increase in PE, although both increase in net amounts in the rat and human brain^36–38^. A rapid quantitative increase in arachidonic acid and DHA is also known to occur during the first 15 days of life, translating to an accumulation of more than 50% of adult rat brain content of these fatty acid^19^. Of note, myelination begins a little later than synaptogenesis starting d12 postnatal in the rat, resulting in an increase in brain 20:1 and 24:1 fatty acids, which are both prominent fatty acids in myelin. The analysis of the latter fatty acids is however quantitatively challenging given the difficulty in separating myelin-associated fatty acids from fatty acids in other brain tissues^19^.

Our findings suggest adding MFGM to formula may reduce lipid class differences between mother-reared and standard formula-fed animals towards the end of the exclusive milk or formula feeding period. The trend towards higher brain PE, significantly higher PS, and a significantly lower PC:PE ratio at d18 in the non-supplemented animals compared to the mother-reared group is intriguing, as higher PE and PS per unit brain weight are usually positive markers of brain development in the rat and the human^14,37^. Regardless, adding MFGM to the formula seemed to decrease these differences, for which the functional implications on the brain merit further investigation. Changes in brain phospholipid composition have been reported in individuals with anxiety disorders, depression, and cognitive impairment^39,40^. Although Sph was significantly higher in the MFGM− group compared to the other groups at d10, the variability in Sph concentrations within the mother-reared and MFGM+ groups was high as evidenced by the large standard deviations relative to their means. The variability was confirmed by re-analyzing the brain samples and is unlikely due to the analytical method used. Future steps need to investigate whether Sph differences do exist, and if so for which Sph sub-groups, along with other development-sensitive lipids such as gangliosides, cerebrosides and plasmalogens^4,7,21^.

It is also known that DHA increases in the rat (and human) brain during development and is primarily present in gray matter PE and PS^14,19–21,35^. We could not accurately analyze fatty acid composition of brain PS because of the low PS concentrations in the brain samples. However, we found that DHA concentrations in PE at d18 were remarkably similar between MFGM+ and MFGM− groups, at ~16.5%. We calculated indirect estimates of net DHA in brain PE and did not find significant differences due to MFGM addition in formula (MFGM+: 39.4 ± 5.69 µg DHA in PE/50 mg brain wet weight; MFGM−: 33.9 ± 11.2 µg DHA, *P* > 0.05). At least two possibilities might explain these findings: The first is that we did not have enough statistical power to detect significant differences in brain content of PE and net DHA in brain PE between the artificially reared groups. The second is that there is no difference in net PE DHA accumulation in the artificially-reared rats due to feeding MFGM during early development. Conducting these experiments with a larger sample size would be an important extension of the present study to understand our findings.

How MFGM supplementation alters brain lipids may be multifactorial. MFGM supplementation provided phospholipids, estimated at 3.5 mg/day which is consistent with daily phospholipid intake from rat milk^41^. Compared to the fatty acids from vegetable oil, fatty acids from the phospholipids are minimal. It is unlikely that any effects on brain lipids are due to the provision of phospholipid fatty acids. Indeed, bovine milk from which the MFGM supplement is derived is very low in DHA^4,7^. Nonetheless, dietary phospholipids not only act as vehicles to deliver fatty acids into the digestive tract. Their role extend to include the supply of building blocks (e.g. phospholipids themselves or their head group molecules such as choline and ethanolamine) for the synthesis of plasma lipoproteins that deliver diet and liver-derived fatty acids to the brain. Stable isotope tracer studies in neonatal piglets and baboons show higher efficacy of dietary phospholipids than triglyceride-bound DHA for DHA brain accretion^10,42^. We found no difference in brain DHA concentration with MFGM supplementation. Future studies may address whether MFGM-derived phospholipids play any role in endogenous DHA (originating from liver or adipose tissue) accretion in the brain using more functional measures. Work by Rapoport and colleagues^43,44^ stimulates interest into examining brain function through more functional measures, such as brain DHA turnover rather than net DHA content. For instance, when DHA supply to the brain is limiting, DHA turnover in the brain decreases such that net amounts of DHA are preserved^43,44^ potentially at the expense of altered DHA-related functions (eg. signal transduction for neurotransmitter release)^45–47^. Higher plasma concentrations of DHA in the MFGM-supplemented animals compared to non-supplemented controls might indicate a role of MFGM in modulating DHA supply to the brain. Our study did not address the effects of adding DHA and arachidonic acid to the formula in addition to adding MFGM. However in a recent extensive study by Aidoud *et al*.^21^, adding DHA and arachidonic acid to formula in which a mixture of plant-based and dairy-based lipids was used, resulted in the closest brain DHA lipidome composition to that of mother-reared controls. It would be interesting to explore whether the dairy-based lipid blend included MFGM and to determine whether a group supplemented with both MFGM and DHA would further close the gap in reflex development between formula-fed and mother-reared pups.

In addition to MFGM being a source of phospholipids, there is the possibility that the closer PC:PE ratio and PS in the MFGM+ group to the mother-reared group at d18 is secondary to indirect effects such as those mediated by the gut microbiome. Our collaborators have recently shown that MFGM supplementation at d18 restores normal development of the gut epithelium and microbiome in artificially-reared neonatal rats. Broadly, changes in the gut microbiome result in changes in brain neurotransmitters in rodents, although the underlying mechanism (s) are not clear^48,49^. One future line of inquiry is to investigate whether and possibly how the gut-brain axis is involved. Finally, MFGM provides bioactive proteins such as xanthine oxidase, lactadherin, and butyrophilin which have antimicrobial and immunomodulatory properties^6^. Investigating the cellular effects of these compounds on brain development is beyond our area of expertise but is certainly one of potential biological importance.

Several brain metabolites differed between MFGM+ and MFGM− animals mainly on d13 and d18. Without reference data at d13 from the mother-reared group, we are cautious in inferring that MFGM in formula helps “normalize” brain metabolite composition. However, for four metabolites (threonine, glutamine, inositol, and glycerol-3-phosphate), which differed between MFGM+ and MFGM− groups at both d13 and d18, area counts at d18 were reduced with MFGM feeding and brought closer to the reference area counts of the mother-reared group. Glutamine, the precursor of the excitatory neurotransmitters glutamate and aspartate, together with the inhibitory neurotransmitter gamma-aminobutyric acid, ensures adequate glutamate turnover in the brain and protects against excitotoxicity^50,51^. Little is known about the functional roles of threonine in the brain and its physiological brain concentration is dependent on uptake from plasma^52,53^. Inositol functions as a cerebral osmolyte and is a key signaling molecule, as a component of PI and inositol phosphates^54,55^. The inositol triphosphate (IP3)/Ca^2+^ signalling pathway is well-known to control several cellular processes, which in the brain have been implicated in synaptic plasticity^56^. Glycerol-3-phosphate is an intermediate in glycolysis and precursor for *de novo* synthesis of PL. Several questions for future studies remain: Are these metabolites decreased with MFGM feeding due to increased use or decreased need? Are differences in metabolite concentrations due to altered brain metabolism or altered delivery of precursors across the blood-brain barrier? More importantly, do these metabolite differences have functional relevance to the human brain?

Because this research was the first to explore differences in brain lipids due to MFGM feeding in artificially-reared rats, it was not possible for us to conduct power analysis and make assumptions about effect size and standard deviations. We therefore used a less robust method to determine sample size, based on law of diminishing return^57^. Using at least 6 animals per group would result in an “E” value of 15 [(6 animals × 3 groups)- 3 groups = 15] which lies between 10 and 20 and is therefore considered an adequate sample size for exploratory research. Of note, “E” is the degree of freedom of ANOVA across groups, and values of E less than 10 would indicate that adding more animals per group might increase the likelihood of obtaining significant results^57^. More robust power analysis may be used in the future to design studies that reduce the risk for type 2 error associated with inadequate sample size. Additionally, risk for type 1 errors should be taken into account when conducting multiple comparison analyses.

Taken together, this study demonstrates that several milestones in reflex development of formula-fed rat pups are reached at ages closer to those of mother-reared pups when MFGM is added to the formula. Our lipidomics and metabolomics data suggest that adding MFGM to formula may reduce differences in brain phospholipid and fatty acid composition between mother-reared and formula-fed pups, which may have important functional implications for neurodevelopment. However, future studies are needed to confirm our findings using a larger sample size. While bovine MFGM is already added to several infant formula products in the US and China, largely driven by a few intervention studies using different MFGM products and showing short-term improvement in cognitive and immune function^8^, we encourage basic researchers to help address more completely what the short-and long-term risks and benefits of bovine MFGM are and what mechanisms might underlie the effects that are observed.

## Methods

### Animals and provision of formula

All animal procedures were conducted as approved by the Animal Care Committee of the University of British Columbia and conformed to the guidelines of the Canadian Council on Animal Care. Pregnant Sprague Dawley rats at 10–13 days of gestation (Charles River Laboratories; Wilmington, MA, USA) were housed individually in a temperature and humidity-controlled animal facility at the BC Children’s Hospital Research Institute with a 12-hour light: dark cycle and *ad libitum* access to water and food (commercial rodent chow; Harlan-Teklad; www.harlan.com). At day 5 postnatal, rat pups from each litter were randomly assigned to two formula supplementation groups (formula with 6 g/L bovine MFGM or formula without MFGM), with age-matched mother-reared littermates used as reference group^13^. The volume of formula to be fed (ml) was determined daily as 25% of body weight (g). For example, a 30 g pup in the MFGM+ group would be fed 7.5 ml/d formula which would deliver 45 mg/d MFGM. Briefly, formula-fed rats were anaesthetized using halothane, and PE-10 silica tubing cannulas were inserted into the stomach. Gastric cannulas were connected to PE-50 silica tubes attached to micro-peristaltic pumps (Ismatec-IPC; Wertheim, Germany) via cassettes, as previously described^34^. Pumps delivered formula for 10 min in a 30-min cycle (i.e 10 min feeding and 20 min rest), repeatedly for 24 hours a day. Pups were maintained in 500 ml plastic cups, containing corn cob and paper fiber (Biofresh, Absorption Cor) as contaminant-free bedding, and floated on an incubated water bath, maintained at 40–42 °C. Sample size refers to the number of individual pups in each group. We allocated one pup from each litter to each group and for each time point. For example, for a particular litter, we would allocate one pup for the MFGM+ group at d10, one for d13, one for d15 etc. From that same litter, we would also allocate one for the MFGM− group at d10, one for d13 and so on. Any additional pups from one litter would be left with the mother.

### Animal diets

The two milk formulas were prepared as nutritionally adequate rat milk substitutes, following previous methods^41^. Artificially-reared animals either received a formula without MFGM, in which fat was derived from vegetable oils or formula with 6 g/L of bovine MFGM (Lacprodan® MFGM-10, Arla Foods Ingredients, Denmark)^13^. Lacprodan® MFGM-10 provided PE, PI, PS, PC and Sph at 2391 ± 139, 730 ± 46.0, 218 ± 14.0, 1742 ± 98.0, and 1227 ± 51.0 µg/100 mg respectively (based on 4 replicate analyses in our lab). Total fatty acid composition was not different between the MFGM+ and MFGM− formulas, as the additional phospholipids in the MFGM powder itself contributed very minimally to total fatty acids^7^. Of note, Lacprodan® MFGM-10 also contains gangliosides, sialic acid, immunoglobulins, and lactoferrin as claimed by manufacturers. Formula was stored at −20 °C, and thawed formula was mixed with a polytron before use.

### Assessment of physical features maturation and reflex development

Assessment of maturation of physical features and reflex development were carried out twice a day around 9:30 am and 3:30 pm, starting day 5 and until a milestone is achieved, according to Smart and Dobbing^22^. Maturation age refers to the day when a particular feature or reflex was observed/achieved for the first time. The observed physical features were: unfolding of the external pinnae of both ears to the fully erect position, lower incisor eruption with a visible and palpable crest, and opening of both eyes. The following reflex tests were performed:

#### Palmar grasp

The pup is held more or less vertically in one hand with the thumb pressing on the abdomen and chest to hold the pup still while the fingers are supporting the pup’s back and hind-quarters. When the forepaw is extended straight, the pup’s forepaw is gentled touched using a cotton swab and a palmar grasp is observed. The disappearance day of a palmar grasp is registered.

#### Negative geotaxis

The pup is placed head downwards on a 20° slope. Maturation day is the day when the pup turns upright by 180° from starting position within a 90-sec test period.

#### Cliff avoidance

The pup is placed at the edge of a table and is given 30 seconds to avoid the cliff and turn backwards. Maturation day is when the pup withdraws the head and both fore feet from edge within 30 seconds.

#### Ear twitch

The right ear is gently touched using a cotton swab. *Maturation day is when an ear twitch is first observed*.

#### Eyelid twitch

The right eyelid is gently touched using a cotton swab. *Maturation day is when an eyelid twitch is first observed*.

### Tissue collection

Rat pups were anesthetized using isoflurane, blood was collected by cardiac puncture, then pups were euthanized by cutting through the diaphragm at 10, 13, 15 and 18 days postnatal for formula-fed groups and at 5 (baseline on cannulation day), 10, 15, and 18 days postnatal in the mother-reared group (4-5 pups/group/time point). For logistic and resource limitations, we could not collect data from the mother-reared group at day 13. The cerebrum was rapidly removed, weighed, flash frozen in liquid nitrogen and stored at −80 °C until analyzed.

### Brain lipid analysis

Total lipids were extracted from homogenized frontal lobe, then lipid classes (free cholesterol, PE, PI, PS, PC and Sph) were separated using a high performance liquid chromatograph (HPLC) with a quaternary linear gradient solvent system, and quantified with an evaporative light-scattering detector^58^. PE was recovered using a fraction collector, then fatty acids were separated and quantified as their respective methyl esters by capillary column gas liquid chromatography (GLC)^59^.

### Brain metabolomics

50 mg brain tissue was homogenized in 2.25 ml saline and lipids were extracted using 1:2 methanol:chloroform solution. The aqueous phase was dried using a Speedvac centrifugal concentrator. The sample was centrifuged, supernatant removed and evaporated to dryness with a speedvac. Derivatization was performed in 2 steps, first with 100 µl methoxylamine HCL in pyridine (20 mg/ml), then with 75 µl MSTFA, following the protocol of Vallejo *et al*.^60^.Targeted metabolomics was performed by gas chromatography-mass spectrometry (GC-MS) using a Waters Quattro micro ^TM^-GC mass spectrometer (Waters, Milford Massachusetts, USA). A 30 m * 0.25 mm * 0.25 um film thickness column (Agilent HP5MS), with helium as carrier gas was used, and samples were analyzed under the following set conditions: injected sample volume of 1 µL; injector mode as splitless; flow rate of 1.5 ml/min; injector temperature of 250 °C, temperature gradient held at 80 °C for 2 min, then increased to 312 °C at a rate of 8°/min and held at 312 °C for 17 min; total analysis time of 48 min. Mass spectra were acquired between m/z 75–650 at a rate of 120 scans/minute. The mass spectra for each peak was obtained using MassLynx software (version 4.1; Waters Corporation, MA,USA), then used to identify the nature of the metabolites using the online NIST MS library (version 2.0). The nature of identified metabolites was confirmed by comparing their retention times and mass spectra to those of their respective authentic standards.

### Statistical analysis

Statistical analyses were performed using SPSS software (version 20 for Mac OS X) with the level of statistical significance set at *P* < 0.05. Normality of data distribution was tested, where possible, using Kolmogorov-Smirnov test. Differences between means across the three groups were determined using one-way analysis of variance (ANOVA), followed by *post hoc* LSD test where appropriate.

## Electronic supplementary material

Supplementary tables
