# Supplementary material for: Milk Fat Globule Membrane Supplementation in Formula-fed Rat Pups Improves Reflex Development and May Alter Brain Lipid Composition
Source: Sci Rep. 2018 Oct 15;8:15277. doi: 10.1038/s41598-018-33603-8 (PMC6189118; doi:10.1038/s41598-018-33603-8)
Supplement: Supplementary file 1 — Supplementary tables [file 41598_2018_33603_MOESM1_ESM.pdf]

Milk Fat Globule Membrane Supplementation in Formula-fed Rat Pups Improves Reflex Development and May Alter Brain Lipid Composition

Sara Moukarzel[1], Roger A. Dyer[2], Cyrielle Garcia[4•], Alejandra M. Wiedeman[4•], Guilaine Boyce[4], Joanne Weinberg[3], Bernd O. Keller[4], Rajavel Elango [4,5*] and Sheila M. Innis[4‡]

Supplementary Table 1. Total body and brain weights of mother-reared group, group fed formula with MFGM (MFGM+) and group fed formula without MFGM (MFGM-)

		Mother-reared *n*=6	MFGM+ *n*=7	MFGM- *n*=7
**Days postnatal**				
d5				
	Body	16.0 ± 0.47	14.8 ± 0.69	14.4 ± 0.29
	Brain[1]	0.54 ± 0.04	-	-
d10				
	Body	24.3 ± 1.44	21.1 ± 0.12	20.9 ± 1.15
	Brain	0.80 ± 0.04	0.68 ± 0.02	0.88 ± 0.04
d13				
	Body	NA	34.8 ± 1.90	30.3 ± 6.54
	Brain		0.92 ± 0.01	0.93 ± 0.02
d15				
	Body	41 ± 1.56	36.4 ± 2.98	39.1 ± 4.28
	Brain	1.25 ± 0.18	0.94 ± 0.03	1.03 ± 0.02
d18				
	Body	38.9 ± 2.54	37.9 ± 0.45	36.1 ± 2.68
	Brain	0.92 ± 0.01	1.05 ± 0.03	1.00 ± 0.02

means ± SEM in g; [1] values correspond to cerebrum wet weight
NA, not available; No significant difference between groups using ANOVA.

Supplementary Table 2. Maturation age (days) for physical features and reflexes of mother-reared rats, rats fed formula with MFGM (MFGM+ group) and rats fed formula without MFGM (MFGM– group)

	Mother-reared (*n*=6)	MFGM+ (*n*=7)	MFGM– (*n*=7)
**Physical features**			
Ear unfolding	8.33 ± 0.52 [a]	9 [b]	9 [b]
Incisor eruption	8.67 ± 0.82 [a]	8.20 ± 0.84 [a]	8.14 ± 0.69 [a]
Eye opening	13	13	13
**Reflexes**			
Palmar grasp	5	6	6
Negative geotaxis	7.00 ± 0.89 [a]	6.20 ± 0.45 [a,b]	6.00 ± 0.58 [b]
Cliff Avoidance	6 [a]	5.40 ± 0.55 [b]	5 [c]
Ear twitch	6.33 ± 0.52 [a]	8.71 ± 0.76 [b]	9.86 ± 1.07 [c]
Eyelid twitch	6 [a]	6.20 ± 0.45 [a]	6.86 ± 0.38 [b]

Values are mean ± SEM. Differences between groups were analyzed by ANOVA, followed by post hoc LSD test. Values within a row with different letters are significantly different from each other (P <0.05).

Supplementary Table 3. Differences in selected brain lipids between mother-reared rats, rats fed formula with MFGM (MFGM+ group) and rats fed formula without MFGM (MFGM– group)

	d5	d10	d13	d15	d18
	µg/50 mg wet weight				
PE					
Mother-reared	280 ± 56.9	282 ± 33.6	NA	473 ± 31.5	521 ± 69.4*
MFGM+	-	366 ± 62.8	397 ± 48.3	493 ± 107	508 ± 49.1
MFGM–	-	372 ± 135	452 ± 54.6	478 ± 131	612 ± 58.4
PI					
Mother-reared	74.5 ± 15.6	56.1 ± 7.28	NA	88.9 ± 1.17	106 ± 20.3
MFGM+	-	71.3 ± 15.1	81.6 ± 6.38	91.0 ± 34.4	92.0 ± 19.7
MFGM–	-	70.6 ± 23.0	84.0 ± 6.46	89.8 ± 27.9	120 ± 15.9
PS					
Mother-reared	50.1 ± 8.17	36.3 ± 8.79	NA	55.3 ± 0.57	59.1 ± 11.8 [a]
MFGM+	-	48.3 ± 7.09	54.3 ± 3.11	62.2 ± 32.5	67.8 ± 15.6 [a]
MFGM–	-	57.7 ± 18.4	54.1 ± 11.8	71.8 ± 19.6	98.2 ± 11.3 [b]
PC					
Mother-reared	454 ± 99.1	431 ± 51	NA	631 ± 59.2	657 ± 73.5
MFGM+	-	510 ± 71.2	580 ± 59.7	621 ± 92.9	594 ± 65.7
MFGM–	-	515 ± 212	526 ± 56.5	578 ± 120	649 ± 53.7
Sph					
Mother-reared	30.3 ± 12.6	42.1 ±11.5 [a]	NA	60.8 ± 5.29	44.7 ± 8.22
MFGM+	-	34.3 ± 10.1 [a,b]	29.4 ± 5.39	42.3 ± 9.96	35.8 ± 11.0
MFGM–	-	16.3 ± 1.67 [b]	32.1 ± 12.4	31.7 ± 16.2	42.1 ± 11.5
F-Cholesterol					
Mother-reared	165 ± 26.5	225 ± 30.8	NA	441 ± 47.8	501 ± 113
MFGM+	-	275 ± 45.7	367 ± 49.1	414 ± 81.9	408 ± 36.5
MFGM–	-	210 ± 31.1	408 ± 94.9	389 ± 119	499 ± 60.2
PC-to-PE ratio					
Mother-reared	1.56 ± 0.04	1.53 ± 0.02[a]	NA	1.33 ± 0.04	1.27 ± 0.05 [a]
MFGM+	-	1.40 ± 0.09[b]	1.46 ± 0.04[a]	1.28 ± 0.11	1.17 ± 0.04 [b]
MFGM–	-	1.37 ± 0.08[b]	1.18 ± 0.19[b]	1.22 ± 0.11	1.06 ± 0.01 [c]

Values are means ± SD; n=4-6 per group per time point; NA, not available. * PE tends to be significantly different across three groups at d18, by ANOVA (*P*=0.052). Values within a column at any particular time point with different letters are significantly different from each other, using ANOVA followed by post hoc LSD test (*P* <0.05). d, days postnatal; NA, not applicable; F, free; PE, phosphatidylethanolamine; PI, phosphatidylinositol; PS, phosphatidylserine; PC, phosphatidylcholine; Sph, sphingomyelin

Supplementary Table 4. Significant changes in brain metabolites between mother-reared rats (MR group), rats fed formula with MFGM (MFGM+ group) and rats fed formula without MFGM− group) at days 15 and 18 postnatal.

	Fold-differences		
	MR relative to MFGM+	MR relative to MFGM−	MFGM+ relative to MFGM−
**At day 15**			
Threonine	0.22*	0.22*	0.95
Glycine	0.49*	0.52*	1.07
Glutamine	0.52*	0.60*	1.1
Alanine	0.36*	0.36*	1.0
Inositol	0.60	0.57*	0.95
**At day 18**			
Threonine	0.55	0.34*	0.63*
Glutamine	0.64*	0.51*	0.79*
Inositol	0.64	0.52*	0.81*
Glycerol-3-phosphate	0.89	0.32*	0.36*

[1] differences between groups compared by ANOVA followed by post hoc LSD test. *P <0.05.
